# Medical Opinion and Movement

**Published:** 1908-10-17

**Authors:** 


### Medical Opinion and Movement.

A VERY unusual complication of appendicitis is hæmaturia, of which Dr. Seelig, of St. Louis, has seen three instances within quite a short time. The literature on this matter is so scanty that as far as this country is concerned it may be said not to exist. Dr. Seelig points out in the *Annals of Surgery* that the cases may be separated into those of toxic nephritis by systemic invasion from an acute appendicitis, those of direct involvement of one kidney by an appendicular abscess, and those in which the ureter or bladder is kinked by adhesions or perforated by inflammation started by appendicitis. The complication is one which seriously obscures the diagnosis of the real lesion; for it naturally suggests renal colic as the source of the pain and other symptoms. There are few surgeons who have not at some time or another mistaken a case of renal calculus for one of appendicitis, and it now appears that in rare instances it is quite easy to make the opposite mistake, and it is also evident that great care is essential if this is to be avoided. It seems odd that no case of the kind should have been hitherto recorded in Great Britain; possibly some have been, but have escaped Dr. Seelig's search, and possibly the publication of his three cases may produce a crop of hitherto unpublished cases and observations of the same sort. On the Continent several such cases have been recorded, and Dieulafoy especially asserts that nephritis not uncommonly follows acute appendicitis. He further says that the severity of this "Nephrite toxique appendiculaire" is in direct proportion to that of the lesion in the appendix.

ARGUING from the fact that in interstitial hepatitis the function of the liver is disturbed by constricting bands analogous to cicatricial tissue, Moerlin (*Munch. Med. Woch.*) has administered fibrolysin to a patient suffering from cirrhosis of the liver, injecting 2 c.c. of the drug subcutaneously in the region of the liver, two or three times a week. The affection was in the atrophic state with commencing ascites. The general health of the patient was bad, his complexion of a greyish colour, his strength greatly reduced, and his appetite very poor. Potassium iodide was first tried, but its good effects only lasted a few days. Calomel was equally useless. Injections of fibrolysin were next tried, and the patient stood them well. Shortly after the second injection the patient felt better, the amount of urine passed was increased, and the ascites diminished. The patient was subsequently able to leave his bed with his appetite and general health improved. The author found that the liver had regained its normal size. After six injections the patient was able to resume work. In the course of his treatment the patient was given Carlsbad salts and hydrochloric acid. This apparently successful result obtained by Moerlin in the treatment of hepatic cirrhosis by fibrolysin may induce others to try this substance in similar cases. We await with interest the result of further investigations.

SCHWARZENBACH, in an article on the ætiology and treatment of the vomiting of pregnancy, expresses the opinion that this condition is due to an irritability of the stomach, the result of the organ being left empty for too long a period of time. The patient does not eat because she is afraid of vomiting, and she vomits because she does not eat. If this fear of vomiting can be banished she begins to take her food in a satisfactory manner, and cure soon follows. The author thus regards the vomiting of pregnancy as a symptom of inanition, or rather of the absence of some unknown substance usually taken in the food, which the organism makes use of to neutralise the toxins of pregnancy. Most authors consider intractable vomiting to be due to a state of intoxication, and it is possible that the toxins are in part eliminated by the stomach. If this be so, we have an explanation of the violent reaction of this organ. The presence of food in the stomach excites gastric secretion, and the toxins thus diluted are passed on to the bowel by gastric peristalsis. Gastric lavage often does not succeed, partly because it is not undertaken sufficiently early in the morning, partly because too long an interval is left between the lavage and the next meal. In treating a patient search should first be made for any pathological condition of the stomach. If found, this should be treated in an appropriate manner. If none be found, attention should first be paid to the bowels, and then it should be impressed upon the patient that vomiting will only occur as the result of fasting too long. With feeble patients whose appetite is bad search must be made for some article of food which she cares for, but fatty and indigestible substances should be avoided. As soon as the patient has succeeded on several occasions in taking food without subsequently vomiting, it will be found possible to give her milk. During the day food should be taken every two or three hours. A meal should be taken immediately before going to bed, and during the night food should be at hand in case the patient should awake. Breakfast should be taken in bed immediately on awakening. The horizontal position has a favourable effect, especially on morning vomiting. Only a small quantity of food should be taken at each meal. The author has tried this treatment with excellent results upon a large number of pregnant women.

IN a recent number of the *Journal de Médecine et de Chirurgie Pratique*, Dr. J. Castaigne draws attention to a form of chronic nephritis with abundant albuminuria, which fails in any way to respond to the usual treatment for albuminuric nephritis. He cites the case of a patient whom he saw four years ago. At that time the patient was passing 6 grams of albumin daily in his urine. He was put on milk diet, eight pints of milk being given daily, in spite of which he grew weaker, and was unable to do any work. The author then tried the effect of a more abundant dietary, of which raw meat formed the chief part, with the result that the patient quickly recovered and has since been able to follow his employment. In this case there was no dropsy nor any disease of the circulatory system. The urine, which was approximately normal in quantity, always contained from 4 to 6 grams of albumen per diem, and epithelial casts were present. There was never any difficulty in the excretion of urine. The author remarks that a nephritis of this type is of favourable prognosis if the patient submits to a proper dietary. The interest of such cases lies in the fact that they do not respond to a milk diet. It is necessary to replace the large quantities of albumin which the patient loses daily, and since the kidneys perform their functions easily it is possible to order a diet rich in albumen, flesh meat being formally indicated. Such cases must be distinguished with care in order that the proper treatment may be applied.

---

ARTERIO-SCLEROSIS formed the subject of an interesting discussion at the recent French Medical Congress at Geneva. M. Huchard opened with a very able address, and his views are especially worthy of attention. Arterio-sclerosis, he contended, must in the first place be clearly differentiated from atheroma. Atheroma is a local lesion, arterio-sclerosis a general disease with a variety of clinical manifestations, but essentially of the nature of an intoxication. He considers that too much importance is attached to arterial hypertension as a pathological factor and as the *primum movens* of the disease. It is always the result of the intoxication which controls and directs the whole condition. Moreover, in certain cases in which the portal system is markedly involved, giving rise to a condition of so-called " abdominal plethora " with bronchitis, pulmonary congestion and cardiac dilatation, the disease may follow its whole course of evolution accompanied by a fall in arterial tension. M. Huchard is of opinion that the experimental production of atheromatous changes by injections of hypertensive substances, adrenaline and the like, in animals, is of little value for the study of arterio-sclerosis, and no definite conclusions can be drawn therefrom in regard to the pathology of this disease. Such experimental lesions are in no way comparable to the general disease with all its clinical symptoms and complications.

---

THE author has collected statistics in regard to 1,500 cases of the disease. On analysis it appears that the chief ætiological factors are gout, lithiasis, rheumatism, syphilis and tobacco. Alcohol plays a minor rôle, for it is responsible for the condition in only 31 cases. M. Huchard recognises three chief forms of the disease—cardio-renal, myo-valvular cardio-sclerosis, and cardio-bulbar (Stokes-Adams' disease), and he divides the clinical evolution of the disease into four periods—the presclerotic or arterial stage, characterised by arterial high tension of toxic origin; the cardio-arterial with cardiac degeneration; the mitro-arterial; and finally the last stage characterised by complications consequent on cardiac failure. These successive stages are discussed fully, and their appropriate treatment. In the early stage the disease may be controlled by efficient therapeutic measures, a milk or milk and vegetarian diet is advocated and renal insufficiency should be combated by diuretics, and lithiasis by the use of theobromine and thyminic acid. For the arterial hypertension, massage, gymnastics, diuretic waters, carbonic acid baths and vaso-dilator drugs (trinitrine and nitrites) are indicated. For the later stages an exclusively milk diet is insisted upon and particular attention to the renal functions.

---

M. JAQUET, of Basle, also contributed to the discussion and he drew a distinction between arterio-sclerosis in youth, in which a nephritis provoked an atheromatous condition of the arteries, and the disease in advanced age, when it is probably more correct to assume that the renal lesion is a consequence of the vascular affection. The development of the disease is different in the two cases: the young patients with Bright's disease and arterio-sclerosis die of uræmia, whereas the old atheromatous subjects succumb to cardiac failure, pneumonia, or some intercurrent affection. In regard to the much vexed question of arterial hypertension he contends that it is not sufficient in itself to produce the vascular changes of arterio-sclerosis. Although certain causal agents of this affection appear to act by means of the hypertension which they set up, there are others, such as alcohol, which, according to the experimental observations of this author do not affect the blood pressure. One more point in this discussion worth mentioning is the experimental work of M. Bardiet, of Toulouse. He has extracted from normal human urine a substance which, injected into dogs behaves in a manner analogous to adrenaline, causing a rise in blood pressure and rapid variations of the respiratory rhythm. The chemical nature of this substance has not been determined, but he calls it " urohypertensine." It appears to act through the nervous system as its hypertensive effect is abolished by destruction of the spinal cord. The urine of the subjects of arterio-sclerosis is free from this substance, and it is suggested, therefore, that the high arterial tension in this disease is due to the retention of this substance in the system. It exists in abundance in the intestine and is eliminated in the fæces.

---

MORE than a year ago Dr. E. W. Ainley Walker set on foot an inquiry to discover what evidence exists in support of the popular notion that the poison of the bee sting is protective against rheumatism. The question was suggested by certain observations, which pointed to a possible relationship between acute rheumatism and the abnormal production of formic acid in the system under the influence of a streptococcal micro-organism. The evidence collected has now been published and certainly confirms a widespread belief in the efficacy of the bee sting, but rather for chronic rheumatoid affections than for acute rheumatic fever. The most interesting piece of evidence is that of Dr. Terc, an Austrian physician of Marburg, who has carried out the bee-sting treatment for many years and is thoroughly convinced of its beneficial effect. According to Dr. Terc rheumatic subjects do not react in the same way to the bee sting as normal individuals, the usual *secondary* œdematous swelling around the wound failing in the case of the former. It is only after many applications of bee stings that this secondary reaction appears—what Dr. Terc calls the *positive reaction.* Later still a stage of actual immunity is reached, and it is then that the cure from rheumatic symptoms is to be looked for. This therapeutic effect of the bee sting appears due to its formic acid content, and there is a certain amount of evidence in favour of the efficacy of formic acid and the formates for rheumatism. Dr. Couch, of New York, claims to have had considerable success with this drug in the treatment of rheumatic affections. He injects 5 to 8 drops of a 2½-per-cent. solution of formic acid together with an equal amount of 1-per-cent. cocaine solution under the skin in the neighbourhood of the painful joints. The injections should be repeated daily till the pain disappears. It will be interesting to watch for further reports on the bee sting or formic acid treatment of rheumatism.

---

In *La Tribune Médicale* Dr. Albert Fournier proposes to classify the ferments which are capable of transforming sugars into lactic acid under three headings: (1) True ferments, which transform a given sugar into lactic acid in such quantity as fulfils the theoretical chemical equation; (2) paralactic ferments, which split up sugar into alcohol, acetic acid, formic acid, malic acid, lactic acid, etc., but only yield of lactic acid from 50 to 70 per cent. of the theoretical quantity; (3) pseudo-lactic ferments which only yield small quantities of lactic acid—*i.e.* from 1 to 3 per cent. These ferments are found in many substances—milk, cheese, butter, etc. Acid-yielding fermentation delays putrefaction of albuminoid material. This fact explains the anti-putrefactive action of milk ferments and their use in the treatment of intestinal infections. By preference the true ferments alone should be used, as they yield a large quantity of lactic acid.

---

Opinion is still somewhat divided as to the utility of preventive injections of anti-tetanic serum. Drs. Labbé and Lucas-Championnière, at a recent session of the Académie de Médicine, took up the cudgels on behalf of this form of treatment. Their deductions are largely drawn from experiences on the stud-farm of Pin, a place where the ravages of tetanus after the castration of foals had almost taken on an endemic character. The disease disappeared at once after a systematic series of preventive injections had been instituted by the veterinary surgeons. Treatment was also successful in cases where the injections were practised after the onset of the disease. In view of the fact that the serum has proved efficacious in the treatment of animals of divers species, it would be strange if man alone proved refractory to the immunising action of the serum. There is no doubt that the action of the serum is particularly efficacious in veterinary medicine, but this does not discount the fact that the frequency of the onset of the disease has notably diminished in man since preventive injections have become a routine treatment. Such cases as have proved stubborn to this treatment must be accounted for by supposing that the technique of the injections was in some way faulty. The authors therefore consider that it is the duty of the surgeon to use preventive injections in all cases where wounds have been subjected to the risk of infection, and a duty which the patient has every right to have properly carried out.

---

Dr. Leon Desguin has recently published a study of fourteen cases of acute dilatation of the stomach. These include three types of the condition—(1) post-operative gastrectasis, (2) gastrectasis the result of trauma other than by operation, (3) medical gastrectasis. This little-understood condition is perhaps more often the cause of a fatal termination to a malady than is at present thought to be the case, and, seeing that some simple proceeding, such as lavage, is sufficient to alleviate the condition if undertaken in time, the author's observations are worthy the attention of the profession. Patients who are the victims of acute dilatation suffer from meteorism, have a normal or subnormal temperature with an accelerated and sunning pulse. They are also constipated, and do not vomit. When this condition ends in death all that is found at the autopsy is a stomach filled with a brown liquid and gas. In a few cases small ulcerations are found in the mucous membrane of the stomach. It is difficult to point out the cause of the trouble. The author inclines to the opinion that it is due to some inhibitory phenomenon, but thinks there are also predisposing causes at work, such as hyperchlorhydria, infection, ulcers, pyloric constriction, dilatation of the stomach, etc., and, no doubt, in many cases a considerable neuropathic element as well. At the same time he admits as the final cause the contraction of the cardiac end of the stomach, either by simple spasm or by inhibition of those nerves which govern the contractile power of the organ.

---

Whatever be the cause, it is certain that the stomach swells by reason of being unable to empty itself, and the condition is manifested by meteorism of the upper part of the abdomen, by great respiratory embarrassment, by a feeble pulse, pallor, and cyanosis. The prognosis is fatal unless intervention is prompt. The trouble may arise suddenly and with acute manifestations, or may come on insidiously. In the first type washing out the stomach with a warm alkaline fluid may prevent a fatal termination. This treatment should be followed by appropriate drug treatment. Anti-spasmodics should be given to overcome the spasm, which is at the root of the trouble, and cardiac and nervous tonics to overcome asthenia. In cases where the onset is insidious the best treatment consists in trying to re-establish the natural functions of the organ by suitable diet and purgatives, aided if necessary by lavage of the stomach to overcome intestinal infection.

